# Implementation of Simultaneous Multi-Parameter Monitoring Based in LC-Type Passive Wireless Sensing with Partial Overlapping and Decoupling Coils

**DOI:** 10.3390/s19235183

**Published:** 2019-11-26

**Authors:** Juan Ignacio Sancho, Noemí Perez, Joaquin De Nó, Jaizki Mendizabal

**Affiliations:** 1 Department of Electrical and Electronics Engineering, University of Navarra, 20018 San Sebastián, Spain; nperez@tecnun.es (N.P.); deno@tecnun.es (J.D.N.); 2 Centro de Investigaciones Técnicas de Guipúzcoa, 20009 San Sebastián, Spain; jmendizabal@ceit.es

**Keywords:** passive wireless sensing, multiparameter sensing, LC sensors, decoupling coil

## Abstract

Inductor–capacitor (LC) passive wireless sensors are widely used for remote sensing. These devices are limited in applications where multiparameter sensing is required, because of the mutual coupling between neighboring sensors. This article presents two effective decoupling techniques for multiparameter sensing, based on partially overlapped sensors and decoupling coils, which, when combined, reduce the mutual coupling between sensors to near zero. A multiparameter LC sensor prototype with these two decoupling mechanisms has been designed, simulated, and measured. This prototype is capable of simultaneously measuring four parameters. The measurements demonstrate that the changes in capacitance in one individual sensor do not affect the measurements of the other sensors. This principle has been applied to simultaneous wear sensing using four identical wear sensors.

## 1. Introduction

Wireless sensors are useful and desirable in many emerging and industrial applications. As these systems have no physical connections between the sensing components and the processing equipment, they are highly versatile for operating in harsh environments, especially when physical access is difficult. Wireless sensors can operate in an active or passive mode. Active sensors are powered with an internal power source, whereas passive devices receive power remotely. While active sensors can be read from longer distances, they have additional installation and maintenance costs and battery lifetime limitations [1]. On the other hand, passive wireless sensors have limited functionality compared with active ones, but are generally cheaper, easier to implement, and last longer. In addition to this, the implementation of wireless multiparameter passive sensors would increase the density of sensing, and simplify monitoring and processing, which are key issues in the Internet of Things (IoT) world.

There are many types of wireless passive sensors, and a wide range of them are powered magnetically or electromagnetically. An inductor–capacitor (LC) wireless sensor is one of these passive devices, which can remotely sense the parameters of interest, such as humidity, temperature, and pressure [2,3,4]. These sensors use an LC resonant tank to remotely measure a parameter. The sensing principle is usually based on changes in the capacitance as well as in the inductance, which cause a variation in the resonant frequency. The frequency shift is wirelessly measured using an external magnetically coupled coil. LC sensors show some of the advantages of capacitive sensors, such as low power consumption, low temperature drift and good long-term stability, but they also have other drawbacks, like a reduced read range.

Conventional LC sensors are limited in cases of multiparameter sensing [5]. In these cases, more than one LC sensor and readout coil are required. However, an array of separated LC sensors would occupy a large area and could need an individual readout coil for each sensor [6]. Several efforts have been made to achieve compact LC multiparameter sensors. Using only a readout coil and some LC sensors with stacked inductors to reduce the sensing area [7], or dividing the inductor in the sensor into two or more stacked resonant sections [8], results in a shifting or even loss of the individual resonant frequencies as a result of the strong magnetic coupling between the stacked inductors. An LC sensor formed by two partially overlapped embedded inductors with a high overlapping area has also been proposed [9], but the crosstalk among the two inductors is non-ignorable.

The real and imaginary parts of the input impedance in a single LC tank have been employed to simultaneously measure two magnitudes [10], but this method is not feasible with multiple parameters. Additionally, a decoupling scheme for algorithmically solving the crosstalk between two sensors has been proposed by the authors of [11]. This method is limited to two parameters and makes the readout system more complicated. Furthermore, a special inductor structure for stacked inductors based on the partial inductance theory has also been proposed [7,12] in order to minimize magnetic coupling between stacked inductances. This concept achieves a compact design, but the magnetic coupling between each individual LC sensor and the readout coil antenna is drastically reduced; thus, its sensitivity and read range are shortened. Another option is to integrate a switch into the LC sensor system [13], and then making a time-domain measurement. The switch is desirable as a passive element, but this makes the readout and the sensing part more complicated. Recently [14], a branched inductor structure with a sensitive capacitor connected to each branch has been proposed, but the measured magnitudes have to still be analytically decoupled.

**Figure 1:**
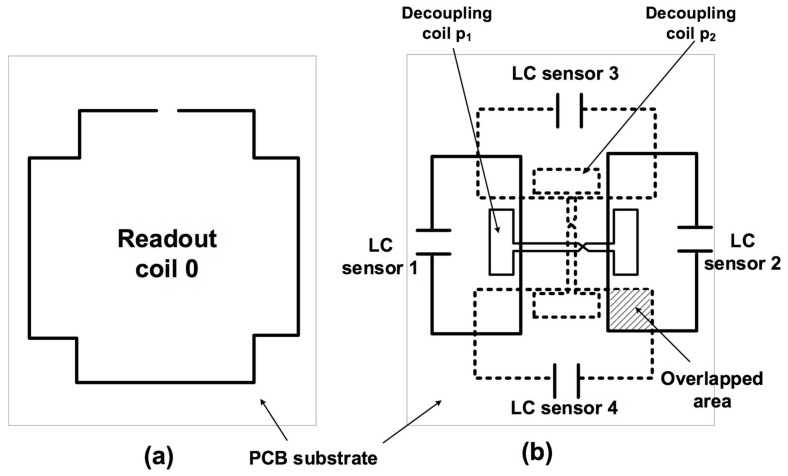
Proposed structure for (**a**) reader coil and (**b**) four partially overlapped rectangular inductor–capacitor (LC) sensors. The reader coil and sensors are printed in a Printed Circuit Board (PCB). Dotted lines correspond to the bottom layer.

In this paper, a configuration of multiple partially overlapped LC sensors is proposed (Figure 1). With this configuration, it is possible to design a specific overlap in order to achieve zero magnetic coupling between adjacent overlapped LC sensors. Furthermore, the use of a decoupling coil as a method for decoupling sensors when partial overlapping is not possible has also been applied. Therefore, a multiparameter sensor with no shifting between the resonant frequencies has been designed. The proposed configuration allows for reading multiple sensors using the same readout coil antenna. 

## 2. Working Principle

**Figure 2:**
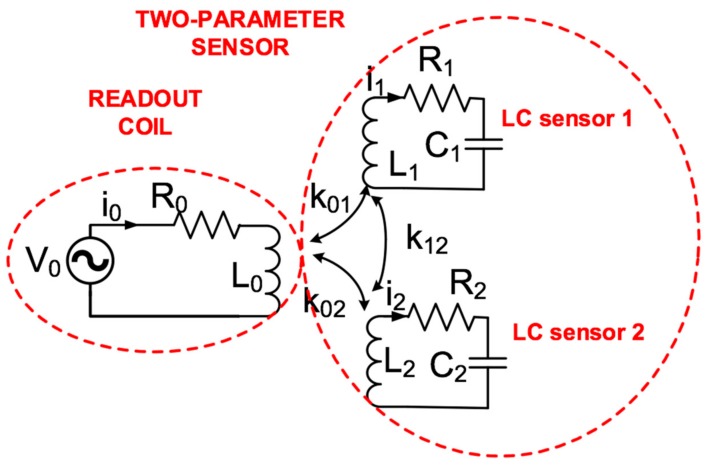
Equivalent circuit and magnitudes for two magnetically coupled LC sensors with a readout coil antenna.

In an LC-type passive multiparameter wireless sensor system, coils form the inductors, and each capacitive sensor is connected to its corresponding inductor, forming a set of LC resonant circuits. The resonant frequencies of a two parameter LC-passive wireless sensor can be determined through its equivalent circuit (Figure 2), taking into account the mutual coupling between coils. Mutual coupling can be described using the coupling coefficients (*k_ij_*) between coils *i* and *j*.

(1) $$k_{ij}=\frac{M_{ij}}{\sqrt{L_{i}\cdot L_{j}}}$$

where *L_i_* and *L_j_* are the self-inductances of coils *i* and *j,* respectively, and *M_ij_* is the mutual inductance between coils *i* and *j*. From Figure 2, for a two coupled sensor system, an approximate solution can be obtained for the two resonant frequencies of these sensors [9], as follows:

(2) $$\omega_{1,2}=\sqrt{\frac{\left(L_{1}C_{1}+L_{2}C_{2}\right)\pm \left(L_{1}C_{1}-L_{2}C_{2}\right)\sqrt{1+\frac{4L_{1}L_{2}C_{1}C_{2}k_{12}^{2}}{\left(L_{1}C_{1}-L_{2}C_{2}\right)}}}{2L_{1}L_{2}C_{1}C_{2}\left(1-k_{12}^{2}\right)}}$$

where *k*_12_ is the coupling coefficient between the two sensors, and *L*_1_ and *L*_2_, and *C*_1_ and *C*_2_ are the inductances and capacitances, respectively. For two strong-coupled LC sensors (i.e., stacked coils), the coupling coefficient (*k*_12_) tends to be 1. In this case, according to Equation (2), the two resonant frequencies tend to be infinite [9], and the following equation is thus used:

(3) $$\omega =\frac{1}{\sqrt{L_{1}C_{1}+L_{2}C_{2}}}\;.$$

However, for two weakly-coupled LC sensors, there are two different resonances. When *k*_12_ is near zero, the two resonant frequencies of the readout coil tend to be independent, as follows:

(4) $$\begin{array}{ccc} \omega_{1}=\frac{1}{\sqrt{L_{1}C_{1}}} & ; & \omega_{2}=\frac{1}{\sqrt{L_{2}C_{2}}} \end{array}.$$

**Figure 3:**
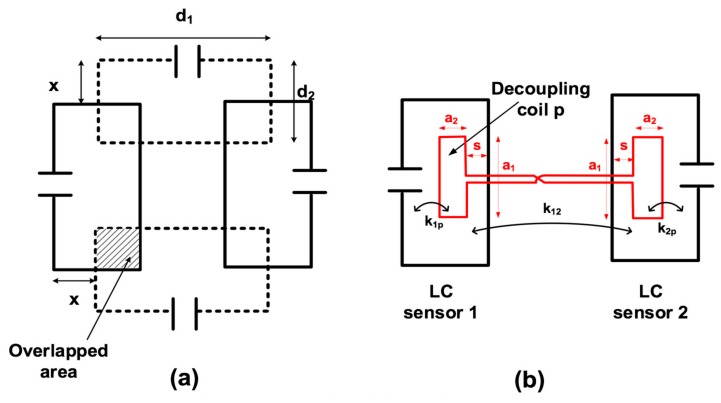
Using (**a**) partially overlapped coils and (**b**) a decoupling coil to achieve zero coupling between two LC sensors.

The coupling coefficient (*k*_12_) between the sensors depends on the geometry of the coils and the relative position between them. There are different ways to have null coupling between two coils [15,16]. In particular, the most effective and simple method for multiparameter sensing is partial overlapping (Figure 3a). A null coupling coefficient between two adjacent coils results in a certain overlap between them. This overlap depends on factors such as the geometry, the thickness of the coil wire, the relative position, and the axial distance between the overlapped coils. When the overlap is not possible, another alternative and simple way is by using an additional decoupling coil [16,17,18,19] that transfers and inverts a small portion of the magnetic flux from one LC sensor to the other (Figure 3b). This inverted flux is able to cancel the magnetic coupling between sensors. Voltage cancellation is possible when an opposite voltage is induced by the decoupling coil, as follows:

(5) $$M_{12}\cdot \omega \cdot j\cdot i_{1}=-M_{2p}\cdot \omega \cdot j\cdot i_{p}$$

where *M*_12_ is the mutual inductance between LC coils 1 and 2, *i*_1_ is the electric current in LC coil 1, *M*_2*p*_ is the mutual inductance between LC coil 2 and decoupling coil p, and *i_p_* is the electric current in decoupling coil p. Neglecting the resistance of the decoupling coil, the electric current in the decoupling coil can be calculated as follows:

(6) $$i_{p}=-\frac{M_{1p}}{L_{p}}\cdot i_{1}$$

where *M*_1*p*_ is the mutual inductance between LC coil 1 and decoupling coil p, and *L_p_* is the inductance of coil p. Consequently, from Equation (5), the following equations are achieved:

(7) $$M_{12}=\frac{M_{1p}\cdot M_{2p}}{L_{p}}$$

(8) $$\frac{M_{12}}{\sqrt{L_{1}\cdot L_{2}}}=k_{12}=k_{1p}\cdot k_{2p}=\frac{M_{1p}}{\sqrt{L_{1}\cdot L_{p}}}\cdot \frac{M_{2p}}{\sqrt{L_{2}\cdot L_{p}}}.$$

This condition must be satisfied in order to achieve null coupling using this method.

The partial overlapping and decoupling coil dimensions can be analytically adjusted using the partial inductance theory [20], but can also be determined using an electromagnetic simulator. Although in practice it is difficult to adjust the magnetic coupling exactly to zero, it can be drastically reduced by these methods. In addition to this, as the resistance of the decoupling coil has been neglected, it will have a small effect in magnetic decoupling, especially at higher frequencies.

## 3. Simulation

**Figure 4:**
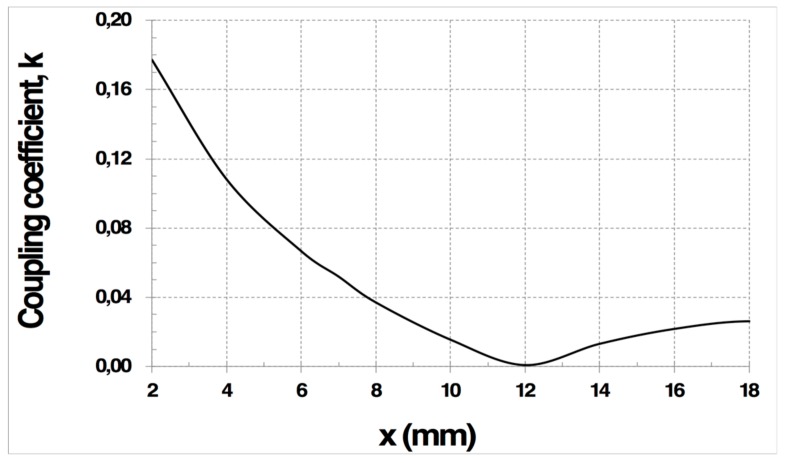
Simulated (CST Studio Suite) coupling coefficient (k) between two overlapped LC sensors as a function of its overlapping x. The x (mm) magnitude is described in Figure 3.

To demonstrate how the magnetic mutual coupling can be suppressed, an electromagnetic simulation using the software CST Studio Suite was carried out. First, a structure of four rectangular sensors without decoupling coils, as described in Figure 3a, was simulated, with d_1_ = 40 mm and d_2_ = 20 mm, in an 0.8-mm thick FR4 substrate. The trace width was fixed to 1 mm. The inductance matrix was obtained as a result. The coupling coefficients between the LC sensors could be easily determined from the inductance matrix using Equation (2). Simulations were carried out in order to determine the optimum overlapping x, which achieved a zero-coupling coefficient between the LC sensors. Hence, an optimum overlap of 11.8 mm was determined (Figure 4). Then, two decoupling coils, p_1_ and p_2_ (Figure 1), were added to the same sensor structure for the simulation. The dimension of s = 0 was fixed (Figure 3b). For these decoupling coils, dimensions a_1_ and a_2_ and the trace width were determined between the non-overlapped coils, in order to satisfy Equation (8). Table 1 shows the final parameters of the four LC sensors and the two decoupling coils.

## 4. Experiments

**Figure 5:**
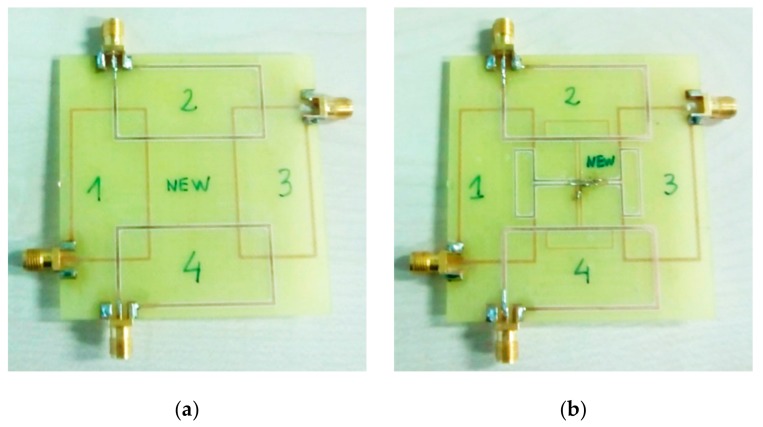
Structure of a four partially overlapped rectangular LC sensors for measuring the s_21_ transmission scattering parameter between them: (**a**) without decoupling coils and (**b**) with decoupling coils.

Two sensor structures (four coils in each) were implemented in a double-sided 0.8 mm-thick FR4 substrate, according to the values in Table 1. One structure had no decoupling coils (Figure 5a), while the other did (Figure 5b). Each coil could be connected to one port of the network analyzer by means of a coaxial cable.

**Figure 6:**
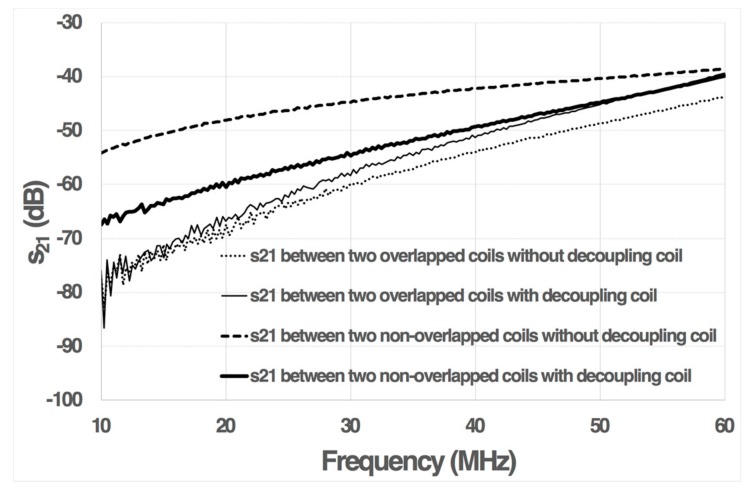
A comparison of the obtained s_21_ transmission scattering parameter between two generic sensors (1 and 2) using partial overlapping and decoupling coils.

The magnetic coupling between two generic coils, coils 1 and 2, from one structure was characterized by means of an Agilent 8714ET RF Network Analyzer, by measuring the scattering transmission parameter, s_21_, between them (Figure 6). If the magnetic coupling decreased between the two coils, the voltage and power transfer between these coils also decreased; and consequently, the s_21_ parameter should decrease. 

Figure 6 shows the s_21_ parameter for two non-overlapped coils without a decoupling coil. Its value remained small (below −40 dB), but higher than the corresponding value for the overlapped coils. Figure 6 also shows that the coupling coefficient between two non-overlapped coils can be reduced to a value comparable to that of two partially overlapped coils, by means of a decoupling coil. Flux cancellation became more difficult at higher frequencies, because of the self-resonance of the coils. Furthermore, the decoupling coil had a small effect in the s_21_ parameter of the overlapped coils at higher frequencies because of its resistance.

**Figure 7:**
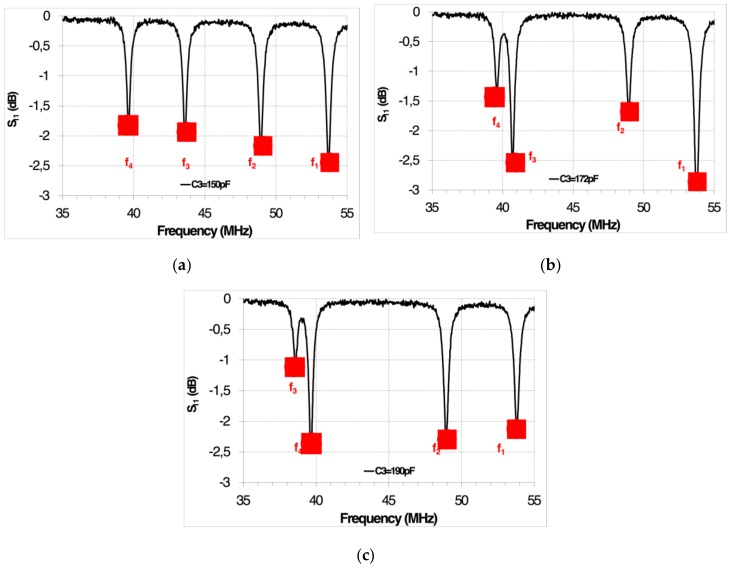
Measured resonant frequencies in the four-parameter LC sensor. The coils are separately connected to four discrete sets of SMD ceramic capacitors (C_1_, C_2_, C_3_, and C_4_). Capacitors C_1_, C_2_, and C_4_ remain unchanged; and consequently, the resonant frequencies (f_1_, f_2_, and f_4_), respectively, remain unchanged. Capacitor C_3_ is changed to values of (**a**) C_3_ = 150 pF, (**b**) C_3_ = 172 pF, and (**c**) C_3_ = 190 pF, changing the resonant frequency (f_3_).

After this experiment, a set of discrete SMD ceramic capacitors, with values of C_1_ = 100 pF, C_2_ = 118 pF, C_3_ = 150 pF, and C_4_ = 182 pF, were separately connected to the four designed inductors (Figure 1b), forming four LC resonant circuits. A readout coil with a structure as shown Figure 1a was fixed at 20 mm from the four LC resonant circuits. The resonances of the four-sensor system were simultaneously measured by means of an Agilent 8714ET RF Network Analyzer connected to the readout coil (Table 2). As an example, Figure 7 shows the changes in resonant frequencies when C_3_ changed from 150 to 190 pF in the corresponding LC resonant coil. 

## 5. Application to a Wear Sensor

Finally, four capacitive wear sensors were designed to simultaneously measure the wear in four different positions of abradable blades. As a wired resistive wear sensor has already been implemented [21,22,23], a capacitive analogous version would allow for wireless measurement.

### 5.1. Capacitive Wear Sensor

**Figure 8:**
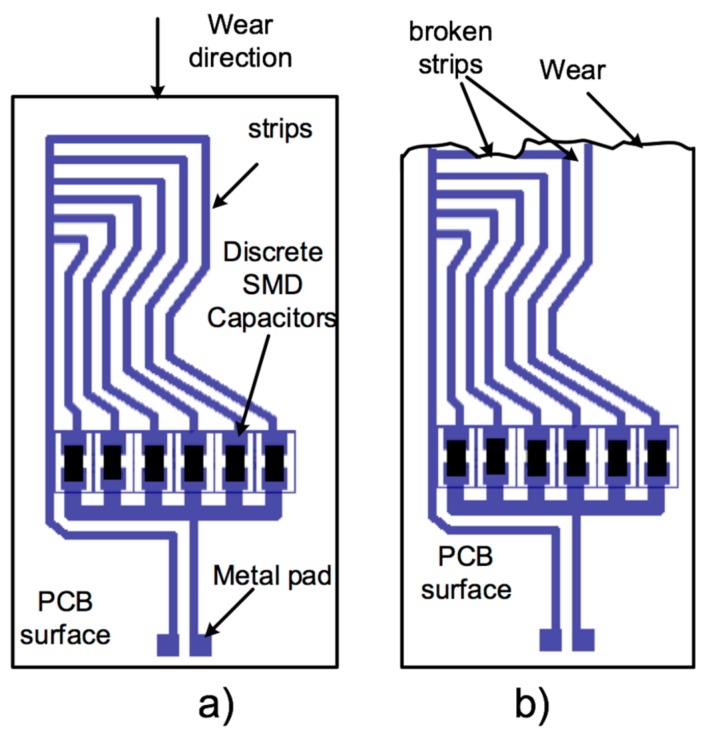
Structure of the capacitive wear sensor: (**a**) before wear occurs and (**b**) when wear occurs.

The working principle of the designed capacitive wear sensor is shown in Figure 8. This sensor was located on a 0.5 mm thick FR4 Printed Circuit Board (PCB), with closely spaced interconnected conductive strips. Each strip was connected to a discrete SMD capacitor, forming a set of capacitors connected in parallel. As shown in Figure 8, six capacitors were connected in parallel. The sensors were rigidly fixed on the surface of an abradable blade. Many materials could be used for the abradable blade, depending on the application. In this case, a carbon fiber abradable blade was used. Furthermore, each individual capacitive sensor was connected from the metal pad to one individual partially overlapped coil (Figure 1b), forming four LC resonant wear sensors. When wear occurred in the blade as a result of contact or friction with another piece, each individual sensor also suffered similar wear. Then, the strips were progressively removed along the wear direction (Figure 8b), causing a smaller parallel capacitance. As a result, the resonant frequency of this particular LC sensor changed.

### 5.2. Wear Measurement

Four capacitive wear sensors were calibrated to provide the same resonant frequency without wear. Assuming that the strips break instantaneously from wear, the resolution of a sensor depends on the center-to-center spacing between the strips. In this case, the distance between the traces was fixed at 1 mm. The discrete capacitor values of each sensor were selected so that the variation of the resonant frequency would be linear with wear. The wear sensitivity was adjusted to 4.5 MHz/mm in order to be robust in front of the discrete capacitor tolerances, parasitic inductance, and resistance as a result of the strips and cabling, as well as the possible interactions between the capacitive sensors when placed close together. 

**Figure 9:**
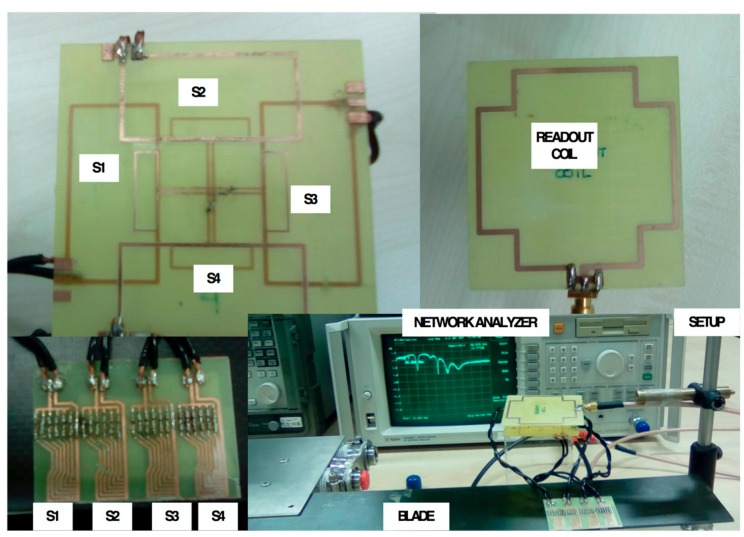
Measurement setup. The four capacitive wear sensors are fixed on the blade and connected with cables to four partially overlapped coils with decoupling coils.

Unlike the previous case in Section 4, the resonant frequencies of all of the sensors without wearing were adjusted to 22 MHz. As the resonant frequencies were equal in all of the sensors, each individual sensor was expected to be more sensitive to magnetic coupling with another sensor. The wear measurement was done in a laboratory using a carbon fiber blade with four sensors (S_1_, S_2_, S_3_, and S_4_) fixed on the blade (Figure 9), and the blade was cut to simulate wear. The distance between the readout coil and the partially overlapped coils was fixed at 6 mm.

**Figure 10:**
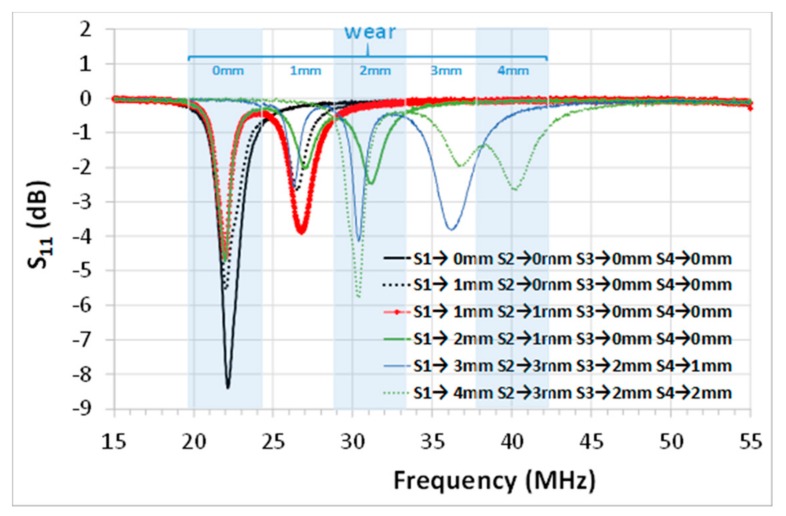
Measured resonant frequencies with four capacitive wear sensors (S_1_, S_2_, S_3_, and S_4_) for different wears. The actual wear values for each sensor are defined in the legend. The resonant measured frequencies are associated with a wear (shown at the top of the figure).

Figure 10 shows the peak resonances obtained for the different values of wear in each sensor, and their corresponding wear. Before the wear occurred (S_1_→0 mm, S_2_→0 mm, S_3_→0 mm, and S_4_→0 mm), the sensors had the same peak resonance at 22 MHz, and consequently, only one peak resonance was measured by the readout coil. When a sensor suffered (S_i_) wear, its respective resonant peak would suffer a frequency shift, but the other sensors provided the same resonant frequency peak at 22 MHz. Then, the readout coil simultaneously read the two resonant peaks. For example, the measured peak resonance was 22.15 MHz with no wear (black solid curve in Figure 10), and the measured peak resonances were 22.02 and 26.5 MHz with 1 mm of wear in sensor S_1_ (black dotted curve in Figure 10). Two similar resonance peaks were observed when comparing one single sensor (S_1_) with 1 mm of wear (black dotted curve in Figure 10, peak resonances at 22.02 and 26.5 MHz) and two sensors (S_1_ and S_2_) with 1 mm of wear (red curve in Figure 10, peak resonances at 21.975 and 26.725 MHz). 

In general, for small wear values, minimal differences were observed between the peak resonances when one or more sensors had the same wear. In any case, this frequency shift should not only be attributed to the magnetic coupling, but also to the tolerance of the SMD capacitors and the parasitic inductance of the cables.

The frequency shift between one or more sensors with the same wear became, relatively, more relevant for higher wearing values (about 2 mm). For example, the peak resonance changed from 31.125 MHz when S_1_ experienced a wear of 2 mm (blue solid curve in Figure 10), to 30.35 MHz when S_3_ experienced a wear of 2 mm (green solid and dotted curves in Figure 10). At these frequency values, the individual sensor capacitance was small; and the parasitic effects due to the inductance of the cable in each individual sensor become more relevant. However, the difference observed between the resonant peaks for the same wear was much smaller than the sensitivity (4.5 MHz/mm). 

The read range was short because of the parasitic cable resistance and small coupling coefficient between the four sensors and the readout coil. Therefore, a balance between the number of parameters to be measured and the read range has to be taken into account, as the number of sensors reduces the read range. Other conventional capacitive sensors have to be studied in the future.

## 6. Conclusions

A novel structure of multiple planar LC sensors with partial overlapping and a decoupling coil is presented in this article. These two techniques allow for relative compactness and multiparameter measurement, allowing for a smaller area in the integration of multiple LC sensors. In addition, they also allow for maintaining near zero coupling between sensors, simplifying the readout system and the sensing system. 

Partial overlapping has been determined using an electromagnetic simulator, in order to minimize coupling. A very low magnetic coupling has been achieved, obtaining an s_21_ parameter below −45 dB, in the range 10–60 MHz. This technique can be used with other geometries, after first carefully determining the appropriate partial overlap. 

The geometry for decoupling coils has also been fixed by using an electromagnetic simulator. Decoupling coils have allowed for a reduction of between 2 and 14 dB in s_21_ for two weakly-coupled coils. Other decoupling geometries can be designed.

A balance between the number of parameters to be simultaneously measured and the magnetic coupling between the readout coil and each individual sensor has to be taken into account, as the more parameters that are measured, the less area each sensor has; thus, reducing the sensitivity and read range.

This system has been applied to four different simultaneously-measuring LC sensors. The experimental results show that the resonant frequencies of non-active LC sensors remain unchanged when the sensing capacitance of an active LC sensor is changed.

This configuration has also been used to successfully monitor wear using four identical capacitive wear sensors. In this case, the parasitic effects due to cabling had to be taken into account, especially at higher frequencies. Therefore, it is preferable to directly mount the capacitive sensors as near as possible to LC coils.

## Figures and Tables

**Table 1:** Dimensions of rectangular coils (LC sensors S_1_, S_2_, S_3_, and S_4_) and decoupling coils (p_1_ and p_2_). All of the dimensions are defined in Figure 3. As a result of the symmetry, the four rectangular coils and the two decoupling coils have equal inductive parameters.

Rectangular Coils		Decoupling Coils p_1_ and p_2_	
d_1_ (mm)	40	a_1_ (mm)	18
d_2_ (mm)	20	a_2_ (mm)	5.5
x (mm)	11.8	s (mm)	0
Self-inductance of coil i L_i_ (nH)	91	Self-inductance of decoupling coil p Lp (nH)	73.4
Mutual inductance between overlapped coils i and j M_ij_ (pH)	51	Mutual inductance between coil i and decoupling coil p M_ip_ (nH)	7.8
Mutual inductance between non-overlapped coils i and j M_ij_ (pH)	825	Coupling coefficient between coil i and decoupling coil p k_ip_	0.0954
Coupling coefficient between non-overlapped coils i and j k_ij_	0.0091		
Trace width (mm)	1	Trace width (mm)	0.6
Substrate	FR4	Substrate	FR4
Subtrate thickness (mm)	0.8	Subtrate thickness (mm)	0.8

**Table 2:** Measured resonant frequencies (from the minimum of s_11_) changing C_1_ and C_3_ values. The values of C_2_ and C_4_ have also been changed, with identical results.

C_1_ (pF)	C_2_ (pF)	C_3_ (pF)	C_4_ (pF)	f_1_ (MHz)	f_2_ (MHz)	f_3_ (MHz)	f_4_ (MHz)
100	118	150	182	53.67	48.92	43.60	39.62
110	118	150	182	51.17	48.90	43.62	39.62
133	118	150	182	46.15	48.95	43.67	39.65
140	118	150	182	45.05	48.92	43.65	39.60
168	118	150	182	40.72	48.87	43.7	39.55
100	118	160	182	53.65	48.90	42.20	39.62
100	118	172	182	53.72	48.90	40.68	39.58
100	118	190	182	53.80	48.92	38.55	39.60
100	118	218	182	53.75	48.90	35.92	39.57
